# 283例系统性治疗的肺癌患者静脉血栓栓塞症的危险因素分析

**DOI:** 10.3779/j.issn.1009-3419.2019.07.03

**Published:** 2019-07-20

**Authors:** 燕娥 刘, 阳春 顾, 福梅 易, 宝山 曹

**Affiliations:** 100191 北京，北京大学第三医院肿瘤化疗与放射病科 Department of Medical Oncology and Radiation Sickness, Peking University Third Hospital, Beijing 100191, China

**Keywords:** 肺肿瘤, 静脉血栓栓塞, 系统性治疗, 下肢静脉曲张, D-二聚体, Lung neoplasms, Venous thromboembolism, Systemic therapy, Lower extremity varicose vein, D-dimer

## Abstract

**背景与目的:**

肺癌患者是静脉血栓栓塞症（venous thrombo-embolism, VTE）的高危人群，合并VTE者具有较高的死亡率。本研究旨在明确接受系统性治疗肺癌患者的VTE发生情况及影响因素。

**方法:**

回顾性分析2016年1月-2018年12月在北京大学第三医院肿瘤化疗与放射病科接受系统性治疗的283例肺癌患者，卡方检验分析VTE与临床特征间的关系，多因素回归分析影响VTE的高危因素。

**结果:**

283例肺癌患者中，VTE发生率为12.01%（34/283）。有下肢静脉曲张组的VTE发生率为50.00%（5/10），显著高于无下肢静脉曲张组的9.89%（27/273）（*P*=0.001）。远处转移患者的VTE发生率为14.05%（26/185），高于带瘤但无远处转移患者的14.00%（7/50），且高于无瘤患者的2.08%（1/48）（*P*=0.024）。肿瘤活动组的VTE发生率为16.93%（21/124），显著高于稳定组的8.18%（3/159)（*P*=0.025）。首次药物治疗前白蛋白 < 35g/L组VTE发生率为22.00%（11/50），显著高于≥35 g/L组的9.87%（23/233）（*P*=0.017）；D-二聚体 > 0.3 μg/mL组VTE发生率为17.93%（26/145），显著高于≤0.3 μg/mL组的5.80%（8/138）（*P*=0.006）。接受PICC的患者上肢静脉血栓的发生率为9.71%（17/175），显著高于未行PICC组的1.85%（2/108）（*P*=0.010）。肺癌病理类型、白细胞、血红蛋白、血小板计数及是否接受抗血管生成药物治疗等因素与VTE的发生率无关。多因素分析显示下肢静脉曲张、低白蛋白血症、D-二聚体升高是VTE的独立影响因素。

**结论:**

有无下肢静脉曲张、血白蛋白和D-二聚体水平或许是预测肺癌患者系统性治疗期间发生VTE更为有效的因子，可进一步建立新的预测模型并进行前瞻性验证。

## Abstract (en)

**Background and objective:**

Patients with lung cancer have high risk of developing venous thromboembolism (VTE), which has been shown to have a significant impact on mortality. This study was to identify the incidence of VTE in lung cancer patients during systemic therapy and to analyze the risk factors associated with it.

**Methods:**

We retrospectively analyzed the cases of 283 patients with lung cancer who received systemic therapy in the Department of Medical Oncology and Radiation Sickness, Peking University Third Hospital, from January 2016 to December 2018. *Chi*-*square* test and multivariate analyses were used to assess the correlation between clinical features and VTE.

**Results:**

Of the patients we observed, 34 developed VTE, with an incidence of 12.01% (34/283). In patients with lower extremity varicose vein (LVV), there was an increase in the incidence of VTE (50.00% *vs* 9.89%, *P*=0.001). The incidence VTE in patients with distant metastasis was higher than that in patients without distant metastasis, and higher than that in patients with tumor-free (14.05% *vs* 14.00% *vs* 2.08%, *P*=0.024). The incidence of VTE in patients with active tumor was also significantly higher than that in patients without it (16.93% *vs* 8.18%, *P*=0.025). Patients with hypoalbuminemia (albumin < 35 g/L) had more VTE events more than those without did (22.00% *vs* 9.87%, *P*=0.017), and patients with an elevated D-dimer level (> 0.3 μg/mL) developed more VTE than those without did (17.93% *vs* 5.80%, *P*=0.006). There were no significant correlations between pathological types, blood cell count before systemic therapy including leukocyte, hemoglobin and platelet, or antiangiogenic drugs and VTE. Multivariate analysis showed that LVV, hypoalbuminemia and elevated level of D-dimer were independent risk factors of VTE.

**Conclusion:**

LVV, serum albumin and D-dimer level may be potential and more effective predictors of VTE in lung cancer patients during systemic therapy. Basing on these factors, new predictive model can be built, and further study to validate its efficacy is required.

静脉血栓栓塞症（venous thromboembolism, VTE）是血液在静脉内异常凝结，使血管完全或不完全阻塞的一种静脉回流障碍性疾病，包括深静脉血栓（deep venous thromboembolism, DVT）和肺栓塞（pulmonary embolism, PE）两种类型^[1]^。VTE的临床症状常常不典型，肿瘤相关VTE发生率约1.3%-12.6%^[2, 3]^，VTE相关死亡率高达12%-30%^[2, 4]^。在美国，合并VTE的肺癌患者平均医疗费用高达84, 187美元，显著高于无VTE的56, 818美元^[5]^。VTE可导致化疗延迟、降低疗效、延长住院时间、增加治疗费用^[6]^。因此，近年来肿瘤相关性VTE日益被人们所重视及认识，已形成专家共识^[1]^，对于早期诊断和预防VTE、降低患者风险、减轻患者及家庭经济和心理负担具有十分重要的意义。

肺癌患者容易发生VTE，约3%-19%的肺癌患者经历过VTE^[7, 8]^。目前关于肺癌VTE的研究多侧重于围手术期患者，针对系统性治疗的肺癌患者VTE的发生情况及影响因素的研究仍较少。因此，本研究通过回顾性分析2016年1月-2018年12月在北京大学第三医院肿瘤化疗与放射病科住院接受系统性治疗的283例肺癌患者，旨在明确全身药物治疗期间肺癌患者VTE的发生率及高危因素，探寻具有较强影响的预测因素，为今后肺癌VTE的早期预防、早期发现和早期治疗提供了理论依据。

## 1 资料与方法

### 1.1 研究对象

通过电子病历查询系统，收集2016年1月-2018年12月在北京大学第三医院肿瘤化疗与放射病科住院的且符合如下标准的肺癌患者。入组标准：病理明确诊断为肺恶性肿瘤，有明确的肿瘤分期，接受系统性药物治疗的患者（包括化疗、酪氨酸激酶抑制剂和抗血管生成药物等）。排除标准：资料不完善者；持续应用抗凝药物治疗的患者。检索到在此期间住院的肺癌患者共计318例，符合上述标准者共283例。

### 1.2 资料收集

采集指标包括患者年龄、性别、身高、体质量、行为状态、既往病史（包括慢性阻塞性肺疾病、高血压、心脏病、糖尿病、血栓、下肢静脉曲张、6个月内接受根治性肺切除手术等）、烟酒史；肿瘤类型、肿瘤分期、是否化疗、治疗方案、首次药物治疗前的白细胞（white blood cell, WBC）、血红蛋白（hemoglobin, HGB）、血小板（platelet, PLT）、白蛋白、肝肾功能、凝血酶原时间（prothrombin time, PT）、纤维蛋白原降解产物（fibrin degradation product, FDP）（0 µg/mL-5 µg/mL）、D-二聚体（0 µg/mL-0.3 µg/mL）等。采集经外周中心静脉置管术（peripheral central venous catheterization, PICC）植入情况，并采集血管超声或CT血管造影结果。计算患者身体质量指数（body mass index, BMI），BMI=体质量（kg）/身高（m）^2^和内生肌酐清除率（endogenous creatinine clearance rate, Ccr）=[（140-年龄）×体质量（kg）]/[0.818×Scr（µmol/L）]（女性按计算结果×0.85）。

### 1.3 评定标准

肿瘤分期依据美国癌症联合委员会颁布的第7版分期标准^[9]^进行评估。行为状态评分依据美国东部肿瘤协作组（Eastern Cooperative Oncology Group, ECOG）评分系统。

肿瘤活动期是指肿瘤确诊后未行抗肿瘤治疗或治疗后疾病进展的患者；肿瘤稳定期是指经抗肿瘤治疗后疾病处于完全缓解、部分缓解或疾病稳定的以及根治性术后接受辅助化疗和随访期间的患者。

DVT诊断依据静脉超声图像或计算机断层扫描（computed tomography, CT）静脉血管造影判断。PE诊断通过CT肺血管造影或肺通气-灌注扫描证实。肱静脉、腋静脉、锁骨下静脉及颈内静脉血栓定义为上肢深静脉血栓。

### 1.4 统计学方法

应用SPSS 22.0统计学软件分析。计数资料采用卡方检验或*Fisher*精确检验；多因素分析采用*Logistic*回归分析。全部统计检验均为双侧概率检验，检验水准α=0.05，以*P* < 0.05为差异有统计学意义。

## 2 结果

### 2.1 一般特征

283例患者符合研究要求，中位年龄为63（19-82）岁， < 65岁的患者居多，占56.89%（161/283）；男性居多，占65.00%（184/283）；肺腺癌占52.65%（149/283）；晚期患者居多，Ⅳ期患者占63.61%（180/283），接受根治性肺切除手术者占16.96%（48/283）；ECOG 0分-1分患者居多，占88.69%（251/283）；接受化疗者居多，占88.69%（251/283），见表 1。

**1:** 283例肺癌患者的临床特征分析 Analysis of clinical features in 283 patients with lung cancer

Clinical characteristics	Number of patients (*n*)	%
Age (yr)		
≥65	122	43.11
< 65	161	56.89
Median age	63	
Range	19-82	
Gender		
Male	184	65.00
Female	99	35.00
ECOG PS		
0-1	251	88.69
≥2	32	11.31
Pathological type		
Adenocarcinoma	149	52.65
Squamous carcinoma	57	20.14
SCLC	60	21.20
Others	17	6.01
Stage		
Ⅰ	13	4.59
Ⅱ	29	10.25
Ⅲ	61	21.55
Ⅳ	180	63.61
Surgery		
Yes	48	16.96
No	235	83.04
Treatment		
Chemotherapy	251	88.69
TKI	32	11.31
ECOG PS: Eastern Cooperative Oncology Group performance status; SCLC: small cell lung cancer; TKI: tyrosine kinase inhibitor.		

### 2.2 VTE组肺癌患者的临床特征

283例肺癌患者中，VTE发生率为12.01%（34/283），VTE发生时距离肿瘤确诊的中位时间为3.0（0.2-49）个月。34例VTE患者中DVT 24例、PE 7例、DVT合并PE 3例。VTE组患者的中位年龄为60（19-78）岁，男性居多，占76.50%（26/34）；处于肿瘤活动期的患者居多，占61.76%（21/34）；症状性DVT患者占75.00%（18/24），上肢静脉血栓占79.16%（19/24）；症状性PE占57.14%（4/7），左肺动脉栓塞占42.86%（3/7）；症状性DVT合并PE占66.66%（2/3），见表 2。接受PICC的患者共计175例，上肢静脉血栓的发生率为9.71%（17/175），显著高于未行PICC组的1.85%（2/108）（*P*=0.010）。

**2:** 34例发生VTE的肺癌患者临床特征分析 Analysis of clinical features in 34 patients with lung cancer and VTE

	Symptoms and signs	Cases	Thrombosis location	Cases
DVT		24		24
	Symptomatic	18 (75.00%)	Upper limb	19 (79.16%)
	Pain	4 (16.67%)	Lower limb	4 (16.67%)
	Swelling	14 (58.33%)	Superior venae cava	1 (4.17%)
	Asymptomatic	6 (25.00%)		
PE		7		7
	Symptomatic	4 (57.14%)	Left PA	3 (42.86%)
	Dyspnea	3 (42.86%)	Right PA	2 (28.57%)
	Swelling of limb	1 (14.28%)	Bilateral PA	2 (28.57%)
	Asymptomatic	3 (42.85%)		
DVT and PE		3		3
	Symptomatic	2 (66.67%)	Right PA and venae cava	1 (33.33%)
	Dyspnea	1 (33.33%)	Bilateral PA and lower limb	1 (33.33%)
	Swelling of limb	1 (33.33%)	Left PA and lower limb	1 (33.33%)
	Asymptomatic	1 (33.33%)		
VTE: venous thromboembolism; DVT: deep venous thromboembolism; PE: pulmonary embolism; PA: pulmonary artery.				

### 2.3 VTE组与非VTE组临床资料的比较

下肢静脉曲张患者VTE发生率为50.00%（5/10），显著高于无下肢静脉曲张患者的9.89%（27/273）（*P*=0.001）。远处转移患者的VTE发生率为14.05%（26/185），高于带瘤但无远处转移患者的14.00%（7/50），且高于无瘤患者的2.08%（1/48）（*P*=0.024）。肿瘤活动期组的VTE发生率为16.93%（21/124），显著高于稳定期组的8.18%（3/159）（*P*=0.025），见表 3。接受抗血管生成药物治疗的患者VTE的发生率高于对照组，但未见明显统计学差异（*P* > 0.05），见表 4。

**3:** 283例肺癌患者VTE组与非VTE组的临床特征分析 Analysis of clinical data in 283 patients with lung cancer between VTE group and Non-VTE group

	Cases (*n*)	VTE (*n*=34)	Non-VTE (*n*=249)	*χ*^2^	*P*
Gender				2.229	0.135
Male	184	26	158		
Female	99	8	91		
Age (yr)				0.059	0.808
≥65	122	14	108		
< 65	161	20	141		
ECOG PS				0.445	0.505
0-1	251	29	222		
≥2	32	5	27		
BMI (kg/m^2^)				0.414	0.520
≥35	3	0	3		
< 35	280	34	246		
COPD				1.330	0.249
Yes	40	7	33		
No	243	27	216		
Heart disease				0.021	0.885
Yes	44	5	39		
No	239	29	210		
Hypertension				0.156	0.693
Yes	116	15	101		
No	167	19	148		
Diabetes				0.000	0.984
Yes	33	4	29		
No	250	30	220		
Cerebral infarction				0.070	0.791
Yes	33	3	30		
No	250	31	219		
LVV				11.609	0.001^*^
Yes	10	5	5		
No	273	29	244		
Surgery				0.101	0.751
Yes	47	5	42		
No	236	29	207		
Smoke				2.141	0.143
Yes	167	24	143		
No	116	10	106		
Alcohol intake				3.325	0.068
Yes	86	15	71		
No	197	19	178		
Pathological type				5.836	0.120
Adenocarcinoma	149	20	129		
Squamous carcinoma	57	9	48		
SCLC	60	2	58		
Other	17	3	14		
Tumor active stage				5.057	0.025^*^
Yes	124	21	103		
No	159	13	146		
Tumor burden				7.422	0.024^*^
Tumor-free	48	1	47		
Tumor burden without distant metastasis	50	7	43		
Distant metastasis	185	26	159		
BMI: body mass index; COPD: chronic obstructive pulmonary disease; LVV: lower extremity varicose veins; ^*^: *P* < 0.05					

**4:** 283例肺癌患者VTE组与非VTE组的治疗相关因素分析 Analysis of clinical factors associated with therapy in 283 patients with lung cancer between VTE group and non-VTE group

	Cases (*n*)	VTE (*n*=34)	Non-VTE (*n*=283)	*χ*^2^	*P*
PICC				0.000	0.993
Yes	175	21	154		
No	108	13	95		
Chemotherapy				0.516	0.472
Yes	206	23	183		
No	77	11	66		
Antiangiogenic drugs				0.126	0.722
Yes	52	7	45		
No	231	27	204		
Dexamethasone				0.017	0.895
Yes	197	24	173		
No	86	10	76		
EPO				0.860	0.354
Yes	13	0	13		
No	270	34	236		
Blood transfusion				0.000	1.000
Yes	10	1	9		
No	273	33	240		
Hemorrhage				3.488	0.062
Yes	12	4	8		
No	271	30	241		
Infection				1.010	0.315
Yes	42	7	35		
No	241	27	214		
PICC: peripherally inserted central catheter; EPO: erythropoietin; Antiangiogenic drugs included bevacizumab, endostatin, anlotinib or apatinib.					

### 2.4 VTE组与非VTE组血液学指标的比较

本研究发现：白蛋白 < 35 g/L组VTE发生率为22%（11/50），显著高于≥35 g/L组的9.87%（23/233）（*P*=0.017）。FDP > 5 µg/mL组VTE发生率为24.49%（12/49），显著高于≤5 µg/mL组的9.41%（22/234）（*P*=0.003）。D-二聚体 > 0.3 µg/mL组VTE发生率为17.93%（26/145），显著高于≤0.3 µg/mL组的5.80%（8/138）（*P*=0.006）。肾功能Ccr < 90 mL/min的VTE发生率为6.66%（12/181），显著低于≥90 mL/min组的21.57%（22/102）（*P*=0.001）。而在WBC≥11×10^9^/L组、HGB < 100 g/L组、PLT≥350×10^9^/L组VTE发生率均呈现出高于对照组的趋势，但均无明显统计学差异（*P* > 0.05），见表 5。

**5:** 283例肺癌患者VTE组与非VTE组的血液学指标分析 Analysis of hematology indexes in 283 patients with lung cancer between VTE group and non-VTE group

	Cases (*n*)	VTE (*n*=34)	Non-VTE (*n*=249)	*χ*^2^	*P*
WBC≥11×10^9^/L				2.942	0.086
Yes	27	6	21		
No	256	28	228		
HGB < 100 g/L				1.559	0.212
Yes	60	10	50		
No	223	24	199		
PLT≥350×10^9^/L				2.990	0.084
Yes	33	7	26		
No	250	27	223		
Albumin < 35 g/L				5.729	0.017^*^
Yes	50	11	39		
No	233	23	210		
Ccr < 90 mL/min				13.773	0.001^*^
Yes	181	12	169		
No	102	22	80		
PT > 12.8 s or < 8.8 s				1.004	0.316
Yes	18	4	14		
No	265	30	235		
FDP > 5 µg/mL				8.725	0.003^*^
Yes	49	12	37		
No	234	22	212		
D-dimer > 0.3 µg/mL				7.584	0.006^*^
Yes	145	26	119		
No	138	8	130		
WBC: white blood cell; HGB: hemoglobin; PLT: platelet; Ccr: endogenous creatinine clearance rate; PT: prothrombin time; FDP: fibrin degradation product; ^*^: *P* < 0.05					

### 2.5 多因素分析

校正是否有肿瘤负荷、是否处于活动期、有无下肢静脉曲张病史、是否合并低蛋白血症（白蛋白 < 35 g/L）、肾功能是否异常（Ccr < 90 mL/min）、FDP是否升高（FDP > 5 µg/mL）、D-二聚体是否升高（D-二聚体 > 0.3 µg/mL）等因素，*Logistic*分析（向前法）表明下肢静脉曲张、低白蛋白血症、D-二聚体升高是VTE的独立影响因素（*P* < 0.05），见表 6。

**6:** 283例肺癌患者VTE发生率的多因素分析 *Logistics* analysis of the incidence of VTE in 283 patients with lung cancer

Variable	Regression coefficient *β*	Standard error	Wald	*P*	Exp(B)	95%CI
LVV (Yes *vs* No)	3.717	1.035	12.900	0.000^*^	41.156	5.413-312.933
Albumin (< 35 g/L *vs* ≥35 g/L)	1.265	0.537	5.546	0.019^*^	3.542	1.236-10.147
D-dimer (≤0.3 µg/mL *vs* > 0.3 µg/mL)	1.512	0.682	4.919	0.027^*^	4.536	1.192-17.259
LVV: lower extremity varicose veins; ^*^: *P* < 0.05						

## 3 讨论

肺癌患者中VTE有较高的发病率和死亡率^[10, 11]^，但关于肺癌在药物治疗期间VTE的发生情况及影响因素尚缺乏报道。本研究中肺癌患者的VTE发生率为12.01%（34/283），与Connolly等^[5]^和Alexander等^[8]^报道的13.9%和19%相近，但显著高于Chew等^[7]^报道的3.0%-3.4%。产生差异的原因在于本研究人群为接受系统性治疗的肺癌患者，且61.83%的患者接受PICC，而Chew等^[7]^研究人群为新诊断的肺癌患者。此外，化疗、靶向治疗和PICC等因素均可增加VTE的发生风险^[13]^。因此，从VTE发生率上来说并不矛盾。

本研究中带瘤、远处转移的患者更容易发生VTE，与既往研究结果类似^[12, 13]^。处于肿瘤活动期的患者VTE发生率为16.93%，显著高于稳定期的患者（8.19%）。其原因可能为：肿瘤组织本身可分泌组织因子、纤维蛋白溶酶原激活剂等促凝物质，使机体处于高凝状态，而肿瘤细胞损伤血管内皮，更容易在血管壁形成血栓^[14]^。本研究发现肾功能异常患者VTE发生率低于肾功能正常者，但多因素分析未提示两者独立相关。在非肿瘤患者中慢性肾脏疾病与VTE风险独立相关^[15]^。但在肿瘤患者中仍存在争议，Kooiman等^[16]^研究发现合并慢性肾脏疾病的肿瘤患者复发性VTE发生率低于肾功能正常的患者，这与我们的结论类似，而在Joanna等^[17]^的研究中，合并慢性肾脏疾病的肺癌患者VTE事件发生率更高，目前机制尚不明确。此外，本研究中PICC组上肢静脉血栓发生率显著高于非PICC组，可能与中心静脉导管置入后对上肢静脉血管内皮的机械性损伤、血流缓慢及增加血小板的聚集有关。

本研究显示下肢静脉曲张、低蛋白血症及D-二聚体是肺癌VTE的独立危险因素。下肢静脉曲张患者由于静脉管壁变薄、内膜损伤、血液淤滞易发生血小板黏附聚集，容易形成血栓，Barsoum等^[18]^也发现静脉曲张是VTE的独立危险因素。低白蛋白血症者血浆胶体渗透压降低，血浆中水分加速流向组织间隙，导致血液粘稠，使血液与血管内皮细胞之间剪切力增加，引起血管内皮细胞损伤，易形成血栓，且影响凝血因子的合成，尤其是纤溶及抗凝系统，导致血液高凝。本研究中单因素及多因素分析均提示低蛋白血症与肺癌VTE显著相关，而有研究^[19]^仅在单因素分析中发现低蛋白血症与肺癌VTE风险增加有关。考虑产生差异的原因为：该研究为病例对照匹配研究，纳入人群较少。此外，本研究中发现异常水平的FDP和D-二聚体与VTE风险增加有关，且D-二聚体升高是VTE的独立预测因素，与既往研究^[19]^一致。尽管临床上D-二聚体成为VTE首要的筛查指标之一，但由于90%的肺癌患者存在血液高凝状态，其特异性仅为50%^[20]^。目前，已有KRS、PROTECHT、CONKO和COMPASS-CAT VTE的预测评分模型^[21-24]^，但上述评分系统缺乏对下肢静脉曲张、白蛋白水平、D-二聚体的考量，因此，进一步建立和完善肺癌VTE的风险预测模型显得非常重要。

本研究是一项回顾性研究，因为不是所有的患者都例行血管超声或CTPA检查，VTE发生率存在低估的可能；此外，本研究纳入样本量偏少，存在选择性偏倚的可能。

综上所述，本研究发现有下肢静脉曲张病史、低白蛋白血症、高D-二聚体是肺癌患者发生VTE的独立危险因素，提示后续预测模型的建立应纳入有无下肢静脉曲张病史、白蛋白、D-二聚体等因素，进一步进行前瞻性临床验证，从而有助于早期识别、早期预防及早期治疗肺癌VTE，对于改善患者生活质量及预后具有非常重要的意义。

## References

[1] Li H, Jiang GN, China National Research Collaborative Group on Venous Thromboembolism in Thoracic Surgery (2018). Perioperative Venous Thromboembolism (VTE) Prophalaxis in Thoracic Cancer Patients: Chinese Experts Consensus. Zhongguo Fei Ai Za Zhi.

[2] Trinh VQ, Karakiewicz PI, Sammon J (2014). Venous thromboembolism after major cancer surgery: temporal trends and patterns of care. JAMA Surg.

[3] Khorana AA, Dalal M, Lin J (2013). Incidence and predictors of venous thromboembolism (VTE) among ambulatory high-risk cancer patients undergoing chemotherapy in the United States. Cancer.

[4] Lyman GH, Eckert L, Wang Y (2013). Venous thromboembolism risk in patients with cancer receiving chemotherapy: a real-world analysis. Oncologist.

[5] Connolly GC, Dalal M, Lin J (2012). Incidence and predictors of venous thromboembolism (VTE) among ambulatory patients with lung cancer. Lung Cancer.

[6] Heit JA, Spencer FA, White RH (2016). The epidemiology of venous thromboembolism. J Thromb Thrombolys.

[7] Chew HK, Davies AM, Wun T (2008). The incidence of venous thromboembolism among patients with primary lung cancer. J Thromb Haemost.

[8] Alexander M, Ball D, Solomon B (2019). Dynamic thromboembolic risk modelling to target appropriate preventative strategies for patients with non-small cell lung cancer. Cancers (Basel).

[9] Goldstraw P, Crowley J, Chansky K (2007). The IASLC Lung Cancer Staging Project: proposals for the revision of the TNM stage groupings in the forthcoming (seventh) edition of the TNM Classification of malignant tumours. J Thorac Oncol.

[10] Wun T, White RH (2009). Venous thromboembolism (VTE) in patients with cancer: epidemiology and risk factors. Cancer Invest.

[11] Li M, Guo Q, Hu W (2019). Incidence, risk factors, and outcomes of venous thromboembolism after oncologic surgery: A systematic review and meta-analysis. Thromb Res.

[12] Ahlbrecht J, Dickmann B, Ay C (2012). Tumor grade is associated with venous thromboembolism in patients with cancer: results from the Vienna Cancer and Thrombosis Study. J Clin Oncol.

[13] Du H, Zhao HL, Li M (2018). Analysis of the incidence of lower extremity venous thrombosis and its related risk factors in admitted patients with lung cancer. Zhongguo Fei Ai Za Zhi.

[14] Campello E, Spiezia L, Radu CM (2011). Endothelial, platelet, and tissue factor-bearing microparticles in cancer patients with and without venous thromboembolism. Thromb Res.

[15] Cheung KL, Zakai NA, Folsom AR (2017). Measures of kidney disease and the risk of venous thromboembolism in the REGARDS (Reasons for Geo- graphic and Racial Differences in Stroke) study. Am J Kidney Dis.

[16] Kooiman J, den Exter PL, Cannegieter SC (2013). Impact of chronic kidney disease on the risk of clinical outcomes in patients with cancer-associated venous thromboembolism during anticoagulant treatment. J Thromb Haemost.

[17] Joanna RM, Marta L, Eliza KR (2018). Evaluation of risk factors and assessment models for predicting venous thromboembolism in lung cancer patients. Med Oncol.

[18] Barsoum MK, Heit JA, Ashrani AA (2010). Is progestin an independent risk factor for incident venous thromboembolism? A population-based case-control study. Thromb Res.

[19] Shen Q, Dong X, Tang X (2017). Risk factors and prognosis value of venous thromboembolism in patients with advanced non-small cell lung cancer: a case-control study. J Thorac Dis.

[20] Mousa SA (2004). Tissue factor/VIIa in thrombosis and cancer. Methods Mol Med.

[21] Khorana AA, Kuderer NM, Culakova E (2008). Development and validation of a predictive model for chemotherapy-associated thrombosis. Blood.

[22] Verso M, Agnelli G, Barni S (2012). Amodified Khorana risk assessment score for venous thromboembolismin cancer patients receiving chemotherapy: the Protecht score. Intern Emerg Med.

[23] Pelzer U, Sinn M, Stieler J (2013). Primary pharmacological prevention of thromboembolic events in ambulatory patients with advanced pancreatic cancer treated with chemotherapy?. Dtsch Med Wochenschr.

[24] Gerotziafas GT, Taher A, Abdel-Razeq H (2017). A predictive score for thrombosis associated with breast, colorectal, lung, or ovarian cancer: the prospective COMPASS-cancer-associated thrombosis study. Oncologist.

